# Quantitative and qualitative motion analysis for early detection of joint damage in haemophilia patients: study protocol for a pilot feasibility study at a German haemophilia treatment centre

**DOI:** 10.1136/bmjopen-2025-108291

**Published:** 2026-03-31

**Authors:** Sarah Schober, Sandra Hansmann

**Affiliations:** 1 Clinic for Paediatrics and Adolescent Medicine, Paediatrics I – Haematology, Oncology, Gastroenterology, Nephrology, Rheumatology, University Hospital Tübingen, Tübingen, Germany; 2 Clinic for Paediatrics and Adolescent Medicine, Paediatrics III - Neuropaediatrics, General Paediatrics, Diabetology, Endocrinology, Social Paediatrics, University Hospital Tübingen, Tübingen, Germany

**Keywords:** PAEDIATRICS, Bleeding disorders & coagulopathies, Physical Fitness, Gait Analysis, Quality of Life

## Abstract

**Introduction:**

Joint bleeds are the most significant complication in haemophilia, leading to functional impairment and reduced quality of life. Repeated bleeding during early childhood can cause severe physical limitations in adulthood. Early detection of joint damage, combined with therapeutic interventions, has a huge impact on mobility, physical health and long-term quality of life.

**Methods and analysis:**

A novel markerless video motion analysis method using deep neural networks will be explored in this single-centre, prospective, longitudinal pilot feasibility study. The study will include children and young adults with haemophilia, as well as age-matched healthy controls. Standardised video recordings, the Haemophilia Joint Health Score (HJHS), clinical data, accelerometer-based physical activity tracking and questionnaires on quality of life and activity levels will be collected at baseline and after 12 months.

For haemophilia patients, individual consultations will be held after each assessment to discuss joint health and possible interventions. Results will be compared with those of healthy participants to assess the method’s feasibility and to explore outcome differences and correlations. The primary focus will be on evaluating the study’s feasibility, practicability, acceptance, possible risks and data quality with regards to a potential future multicentre trial.

**Ethics and dissemination:**

The study was approved by the local Ethics Committee at the Medical Faculty and the University Hospital Tübingen (project No. 188/2023BO1, approval 5 July 2023, protocol version 2.1) and registered at the German Clinical Trials Register (registered 22 September 2023, https://drks.de/search/en/trial/DRKS00032707). Results will be shared at conferences and in peer-reviewed journals.

This pilot study will explore the feasibility, practicability and acceptance of a protocol using artificial intelligence in gait analysis to identify patients at risk of early joint damage and to monitor the functional status of children and young adults with haemophilia in routine care.

**Trial registration number:**

German Clinical Trials Registry (DRKS00032707).

**STRENGTHS AND LIMITATIONS OF THIS STUDY**

- Prospective longitudinal monocentric pilot study evaluating the feasibility, practicability and acceptance of quantitative and qualitative motion analysis in patients with haemophilia.
- Multimodal assessment combining markerless video-gait analysis, accelerometry, physical examination and patient-reported questionnaires.
- Innovative artificial intelligence-based assessment of video-gait analysis to test clinical application for the first time.
- Primary outcomes include recruitment rates, participant adherence and data quality to inform the design of a future multicentre study.
- Limitations include small sample size, single-centre design, high participant burden, potential bias in self-reports, uncertain adherence to accelerometry, and limited statistical power and interpretability due to heterogeneity.

## Introduction

 Haemophilia is an inherited bleeding disorder caused by a deficiency of clotting factor VIII or IX (haemophilia A or B).^1^ Patients, especially with severe haemophilia, experience recurrent joint bleeds from early childhood. Untreated or repeated bleeding into joints often results in haemophilic arthropathy, characterised by chronic pain, reduced mobility and long-term disability.^2 3^

Early diagnosis of joint damage and timely intervention—such as physiotherapy, factor replacement and orthopaedic care—can significantly improve mobility and quality of life.^4^,^6^ Nevertheless, subtle joint impairments often go unnoticed, especially in small children, leading to compensatory gait patterns and reduced physical activity.^4 7^

Physical activity plays a critical role in the development and long-term health of children with haemophilia.^5 8 9^ However, many patients remain less active than their healthy peers due to pain, fear of injury and lack of treatment coordination.^10^

Despite advances in treatment, haemophilic arthropathy remains a major burden, and joint function must be regularly assessed.^9 11 12^ Standard clinical assessments, such as visual gait observation and the Haemophilia Joint Health Score (HJHS),^13 14^ are either subjective or limited in detecting early impairments. While 3D gait analysis offers precise data on joint movement relevant to everyday life, it is costly, time-consuming and impractical for routine care, especially in paediatric settings.^15^,^19^

Recent advances in artificial intelligence (AI) and video technology enable qualitative, cost-effective and markerless video gait analysis using standard cameras. Deep neural networks can identify anatomical landmarks and gait events, enabling automated calculation of joint angles and gait parameters. This method offers a scalable and accessible alternative to 3D analysis, especially in children.^20 21^ Markerless video gait analysis is comparable regarding accuracy, validity and reliability to marker-based 3D gait analysis in spatiotemporal parameters^22^ even in small children.^23^ Additionally, the meta-analysis showed that kinematic parameters for the hip and knee in the sagittal plane are generally valid and reliable. The angles for the ankle joint are, to date, less accurate than marker-based measurements, depending on the method used.^22^

The HJHS, as a standard clinical assessment tool, has limited sensitivity, resulting in high variability and uncertainty in interpreting changes over time.^24^ Therefore, more sensitive diagnostic tools are needed to detect subclinical joint changes before irreversible damage occurs.^24 25^ Markerless video gait analysis might offer a solution, addressing this critical need in haemophilia care by providing objective, non-invasive assessment of joint health.

Patient outcomes in haemophilia are influenced by various factors, including joint health, physical activity and quality of life. This study focuses on physical health and activity as potential contributing factors. Physical activity can be quantitatively assessed using wearable accelerometers that can objectively measure physical activity patterns in daily life, including intensity of activity and sedentary behaviour—factors closely linked to joint health and quality of life.^26 27^ However, studies using accelerometry in haemophilia are limited and suggest lower physical activity levels compared with healthy individuals.^28 29^ In addition, patient-reported outcome measures (PROMs) of daily activity levels and quality of life will be assessed by self-reported questionnaires.

Early detection of joint damage in haemophilia patients can decrease the disease burden. Therefore, markerless video gait analysis could reduce the time and cost to monitor functional joint status, making it suitable for routine clinical use and enhancing treatment management. The presented study aims to facilitate bridging the gap between highly specialised motion analysis methods and practical clinical monitoring in haemophilia patients and will lay the foundation for a future multicentre trial.

### Aim and objective

The aim of this pilot study is to test the feasibility, practicability and acceptance of the procedures for a quantitative and qualitative motion analysis for early detection of joint damage in haemophilia patients and to identify necessary adjustments for a larger, more comprehensive study.

To assess the feasibility of the study protocol, recruitment rates will be estimated and reasons for ineligibility or refusal will be analysed. The practicability and appropriateness of the study procedures and methods will be evaluated based on data on the proportion of usable video gait recordings, participants’ adherence to accelerometry, proportion of missing data, questionnaire completion rate, sensitivity of measurements to detect changes and unexpected problems during assessments. The acceptance of the study will be determined by evaluating the retention and the dropout rate.

This clinical trial has feasibility as its only primary objective. All clinical outcomes are considered exploratory. The main exploratory outcomes are the detection of early movement changes through markerless video gait analysis and the evaluation of joint health using the HJHS. Secondary clinical outcomes are anthropometric indices, clinical parameters, activity metrics from accelerometry and PROMs from questionnaires on daily activity and quality of life.

## Methods and analysis

### Study design and setting

This study follows a prospective, longitudinal design consisting of cases and controls and will be conducted over a period of 36 months (for details in study design, eligibility and interventions see table 1). It is designed as a pilot feasibility study primarily assessing feasibility, practicability and acceptance parameters as well as exploring selected clinical outcomes. All outcome parameters are listed in table 2 and described in detail in the following sections.

**Table 1:** Study design, eligibility and interventions of the study protocol

**Study design**	
Methodological approach	Single-centre, prospective longitudinal pilot feasibility study including cases and controls
Sample size	24 patients (12 severe, 12 moderate haemophilia), 24 matched controls
Timeline	Four appointments for patients within 18 months, two appointments for matched controls within 12 months
Primary objectives	Feasibility
	Practicability
	Acceptance
Exploratory clinical outcomes	Parameters of markerless video-based gait analysis for the detection of joint changes over the course of 1 year
	Haemophilia Joint Health Score HJHS: Haemophilia Joint Health Score, MoMo-AFB: Motorik-Module Activity Questionnaire, EQ5DY5L: EuroQol 5-Dimension Youth 5-Level questionnaire (for children and adolescents), EQ5D5L: EuroQol 5-Dimension 5-Level questionnaire (for adults), Haemo-Qol: Haemophilia quality-of-life, HEP-Test-Q: Haemophilia & Exercise Project-Test-Questionnaire (HJHS)
Secondary clinical outcomes	Anthropometric measurements, clinical data
	Activity parameters from accelerometry
	Activity parameters from questionnaire (MoMo-AFB)
	Quality of life questionnaire (EQ5DY5L, EQ5D5L)
	Additionally, for haemophilia patients disease-adapted questionnaires (Haemo-Qol, HEP-Test-Q)
**Eligibility**	
Participants	Patients at the Department of Paediatrics and Adolescent Medicine of the University Hospital Tübingen aged >18 months suffering from severe or moderate haemophilia A or B or healthy participants matching one of the haemophilia patients
Inclusion criteria	Given informed consent
	Confirmed severe or moderate haemophilia or age- and sex-matched to an included patient
Exclusion criteria	No informed consent/relevant language barriers
	>30 years or <18 months of age
	Inability to walk a 50 m distance
	Control group: serious injury within the last 6 months, neurological, musculoskeletal diseases or joint complaints
**Interventions**	
Interventions during appointment	Anthropometric measurements (length, weight, etc)
	Collection of clinical data (medical history, chronic disease, health impairments, injuries, etc)
	Collection of disease-specific parameters
	Assessment of HJHS (Haemophilia Joint Health Score)
	Recording video gait analysis
	Completing activity questionnaire (MoMo-AFB)
	Completing quality of life questionnaire (EQ5DY5L, EQ5D5L)
	Additionally, Haemo-Qol, HEP-Test-Q for haemophilia patients
	Handing out Actigraph and documentation protocol with a successive 8-day wear-time at home

EQ5D5L, EuroQol 5-Dimension 5-Level questionnaire (version for adults); EQ5DY5L, EuroQol 5-Dimension Youth 5-Level questionnaire (version for children and adolescents); Haemo-Qol, Haemophilia quality-of-life; HEP-Test-Q, Haemophilia & Exercise Project-Test-Questionnaire; MoMo-AFB, Motorik-Module Activity Questionnaire.

**Table 2:** Applied tools and categories in the assessment

Tool	Categories
**Feasibility measurement data**	
Feasibility	Recruitment rates, reasons for ineligibility or refusal
Practicability	Proportion of usable video gait recordings, participant adherence to accelerometry, proportion of missing data, questionnaire completion rate, technical performance, sensitivity of measurements to detect change and unexpected problems during assessments or interventions
Acceptance	Retention rate, drop-out rate
**Clinical measurement data**	
Video-based gait analysis	Spatiotemporal parameters (eg, gait speed, stride length and cadence)
	Kinematic parameters (eg, joint angle trajectories in sagittal plane (hip, knee, ankle))
Accelerometry	Duration of physical activity/resting periods
	Intensity of physical activity
Haemophilia Joint Health Score (HJHS)	Range of motion in elbow, knee and ankle on both sides
	Extent of swelling in elbow, knee and ankle on both sides
	Muscle atrophy
	Crepitation in elbow, knee and ankle on both sides
	Flexion deficit in elbow, knee and ankle on both sides
	Extension deficit in elbow, knee and ankle on both sides
	Joint pain in elbow, knee and ankle on both sides
	Muscle strength
	Global gait (walking, stairs, running, hopping on one leg)
**Medical record data**	
Disease specific parameters	Disease severity
	Factor activity
	Traumas
	Therapy
	Quantity of factor administrations
	Secondary diagnoses
	Medical history and injuries, bleeding events
	Clinical, laboratory chemistry and imaging findings (as far as collected during routine care)
**Patient-reported outcome measures**	
Activity questionnaire (MoMo-AFB)	General and personal data
	Physical activity in general
	Physical activity in age-specific settings
	Physical activity in everyday life
	Physical-sports activity in clubs
	Physical-sports activity in leisure time outside club
	Social support
	Enjoyment of physical activity
	Physical self-concept
	Physical activity environment
Health-related quality of life (EQ5DY5L, adults: EQ5D5L)	Agility/mobility
	Self-care
	General activities (eg, work, study, housework, family and leisure activities)
	Pain/physical discomfort
	Anxiety/dejection
	Current state of health on a visual analogue scale (VAS) between 0 and 100
HEP-Test-Q (haemophilia patients only)	Physical condition
	Mobility
	Strength and coordination
	Endurance
	Body perception
Haemo-QoL (haemophilia patients only)	Physical health
	Feelings
	Self-perception
	Sports and leisure
	Work and school
	Dealing with haemophilia

EQ5D5L, EuroQol 5-Dimension 5-Level questionnaire (version for adults); EQ5DY5L, EuroQol 5-Dimension Youth 5-Level questionnaire (version for children and adolescents); Haemo-Qol, Haemophilia quality-of-life; HEP-Test-Q, Haemophilia & Exercise Project-Test-Questionnaire; HJHS, Haemophilia Joint Health Score; MoMo-AFB, Motorik-Module Activity Questionnaire; VAS, Visual Analogue Scale.

The study will take place at the Haemophilia Treatment Centre at the Department of Paediatrics and Adolescent Medicine of the University Hospital Tübingen, a facility that provides specialised care in haemophilia management. Patients will be recruited from the outpatient clinic and all assessments will be carried out within the University Hospital. The setting allows access to technical infrastructure for video gait analysis recording, accelerometry devices as well as trained clinical staff for monitoring, intervention and follow-up.

### Participants and recruitment

Haemophilia care at the University Hospital Tübingen is provided through separate paediatric and adult divisions. Together, they manage approximately 30 patients with severe and 30 with moderate haemophilia, the majority of whom are children. The sample size for the feasibility pilot study is primarily guided by practical considerations.^30^ With a targeted recruitment rate of 40%, a sample size of 12 participants per group will be feasible. Methodological recommendations for feasibility studies suggest that samples of 12–30 participants per group are sufficient to assess feasibility outcomes such as recruitment rates, retention, data completeness and preliminary parameter estimates for future sample size calculations.^31^,^33^ Since the subgroups of severe and moderate haemophilia will be evaluated separately, the number of cases in each of these two groups is planned to be 12, resulting in a total of 48 participants (24 patients and 24 control subjects).

For each patient with haemophilia enrolled in this study, one healthy subject will be recruited to provide a normative reference dataset. The healthy subjects will be matched to the haemophilia patients based on age, gender, height and body mass. All patients of the Department of Paediatrics and Adolescent Medicine of the University Hospital Tübingen and healthy siblings from the age of 18 months will be able to participate in the study if they fulfil the inclusion criteria and none of the exclusion criteria applies (see table 1). Comparison with age-matched healthy controls will be essential due to the wide age range and heterogeneity of the study cohort. While most participants will be children, young adults will also be included to ensure a sufficient sample size in the moderate and severe haemophilia groups, given the rarity of the condition.

### Timeline

**Figure 1:**
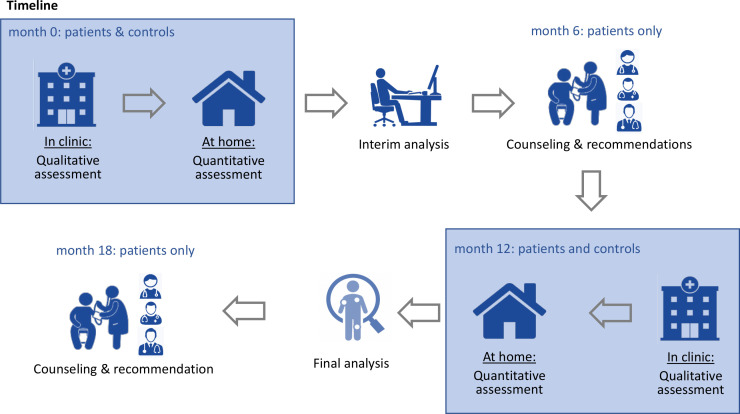
The timing of the measurements and the process of the study.

The timeline of the study protocol is summarised in figure 1. For every study participant, measurement data will be obtained two times in a 12-month period: in month 0 and in month 12. It is expected to include two patients and two healthy participants per month. Based on this estimate, a recruitment period of 1 year will be considered feasible. The total assessment time of the study will be 24 months.

Measurement data collection will take approximately 45 min per patient (video recording 15 min, HJHS 30 min), and the completion of questionnaires will take another 30 min. Subsequently, the wearable activity tracking device will be explained and handed out to the participants to be worn as a regular part of their daily routine for eight consecutive days. Measurement data of haemophilia patients will be collected during routine appointments in the coagulation clinic. Between the two measurement points, individual advice on activity, physiotherapy, muscle strengthening, joint protection and therapy will be provided to the haemophilia patients based on the results of the interim evaluation at month 6. Finally, the second measurement (month 12) will focus on the identification of any measurable changes in the collected data (gait analysis, HJHS, daily activity, questionnaires, etc). Thereafter, the results of all data collected and analysed over the 12-month study period will be communicated to each patient in a dedicated counselling session, including personalised recommendations for best joint health. Furthermore, the aggregated data analysis might provide general recommendations for haemophilia patients to maintain long-term joint health for all patients.

### Interventions and measurements

The interventions and measurements are summarised in table 2 and described in detail in the following sections.

### Assessment of feasibility

Throughout the pilot trial, the number of participants screened and those who are eligible and willing to participate are collected, as well as reasons for ineligibility or reported causes for refusal. To check for the practicability of the study interventions and measurements, the quality of data is reviewed during the entire study, especially the proportion of usable video gait recordings, proportion of missing data from accelerometry and questionnaire completion rates. If necessary, additional descriptions or assistance will be provided for the remaining study in order to improve data quality. Furthermore, the participants’ adherence to the video recordings, accelerometry and the questionnaires is acknowledged and the technical performance, sensitivity of measurements to detect change and unexpected problems during assessments and interventions are recorded. The retention rate and drop-out rate are documented to address acceptance.

### Gait analyses

The recording of standardised videos for the markerless gait analysis will take place in a separate room with a 9.5 m walkway. Walking trials will be captured simultaneously by a sagittal and a frontal camera (AXIS P1375 network camera, 1920×1080 pixels, 50 frames/s). The cameras will be mounted at a height of 50 cm. The frontal camera will be positioned along the walking direction to capture frontal or posterior views. The sagittal camera will be positioned perpendicular (90°) to the walking direction at a distance of 2.8 m from the centre of the walkway.^34^ To counteract distortions caused by the camera lenses at the boundaries of the video frames, only the middle section of the walking distance will be selected for further processing.

The measurements will be taken barefoot with shorts and tight T-shirts; consequently, the ankle, knee joints and hips will be clearly visible. The participants will receive standardised instructions and should walk at a self-selected speed. Three videos will be recorded for each participant, with each video capturing three walking directions (ie, back and forth along the walkway). The total recording will take approximately 15 min.

**Figure 2:**
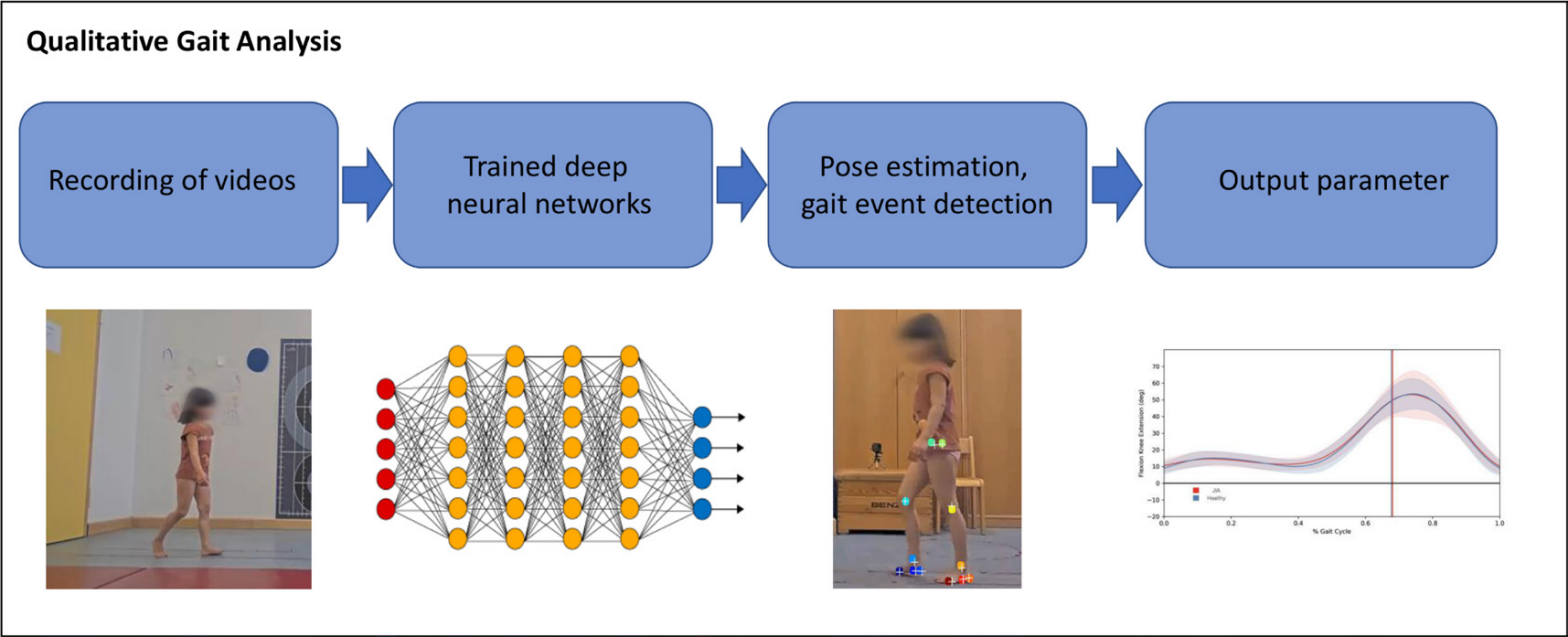
Qualitative gait analysis with standardised videos and evaluation using artificial intelligence.

The cameras will capture participants fully, including their faces. The faces will be made unrecognisable using video editing software before further processing and pseudonymised storage of the videos. The qualitative, markerless gait analysis was evolved by the Children’s Hospital Tübingen and the Cluster of Excellence—Machine Learning for Science at the Eberhard Karls University Tübingen. The method is based on transfer learning with deep neural networks (figure 2) and uses open-source image recognition.

To predict the key points automatically from the recorded sagittal plane videos, a pre-trained Mask R-CNN model is used and a TCFormer model pre-trained on the COCO Whole Body dataset is applied on the cropped images to detect the key points of the human.^35^ Twelve points of interest for 2D motion analysis (shoulders, hips, knees, ankles, heels and toes) are predicted, representing the joint centres and anatomical regions of interest for 2D gait analysis. To automate the identification of individual gait cycles in a given video, a machine learning model, specifically, a Gradient Boosting based classifier, is trained to predict the probability that a given frame is an initial contact event.^36^ The gait analysis is adapted to the special conditions of the analysed age group; proof of principle has been successfully completed in patients with juvenile idiopathic arthritis.^20^

The use of single trials or gait cycles from markerless systems can introduce additional variability in kinematic data.^37^ Therefore, the average of the biomechanical gait data from nine consecutive walking trials at self-selected speed will be calculated, and gait cycle time will be expressed in per cent. The prespecified clinical outcome parameters will be standard spatiotemporal parameters, including cadence (steps/min), stride length and gait speed (m/s) normalised to body height. Additionally, the average joint angle trajectories of the ankle, knee and hip joints in the sagittal plane will be determined.

### Haemophilia Joint Health Score

The HJHS is a clinical score that has been established and validated in haemophilia patients to evaluate joint health and joint changes (table 2). The HJHS assessed by physiotherapists is part of the routine follow-up consultations in all haemophilia patients, regardless of the presented pilot study to evaluate joint health and will take a maximum of 30 min.^13 14^

### Accelerometry

An accelerometer, Actigraph wGT3X-BT (ActiGraph, Pensacola, FL; CE marking EN60601-1-Medical Device General Safety Requirements), will be used to objectively assess the individual physical activity and rest periods of the participants on eight consecutive days during waking hours. The measuring device is equipped with Actigraph’s validated triaxial piezoelectric accelerometer and digital filter technology. Each accelerometer will be initialised with ActiLife 6 Lite version 13.6 (ActiGraph) according to a standardised procedure before being given to the patient.

**Figure 3:**
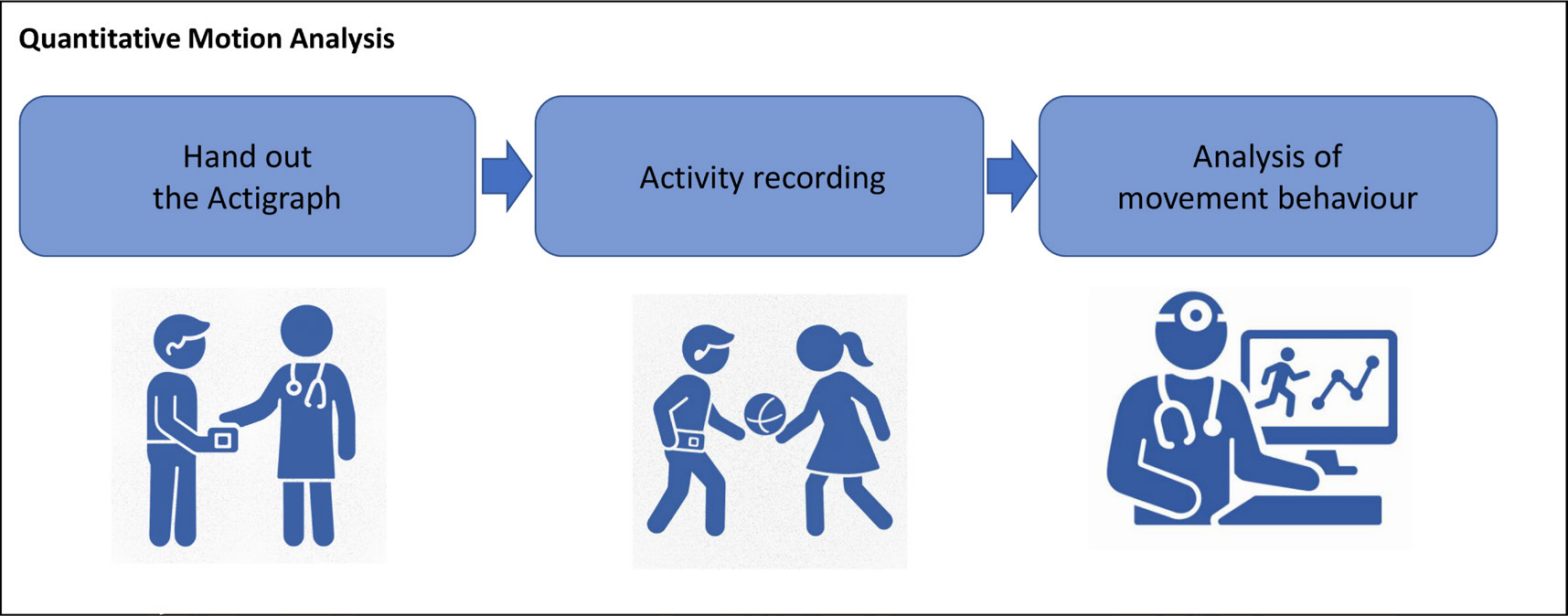
Quantitative motion analysis with accelerometry.

The device will be issued to the participants to take home after the video recording (month 0 and month 12). The participants will be instructed to wear the device on their right hip with a strap. The Actigraph will be taken off for sleeping, bathing/showering, swimming and contact sports. To document no-wear time, the participants will be handed a standardised protocol. Recording will automatically end at midnight on the eighth day and the Actigraph will be subsequently sent back to analyse the recorded raw data (figure 3).

Raw data will be processed with ActiLife 6 full version 13.6, data will be integrated into 1 s epochs as recommended for children and adolescents.^38 39^ Activity intensities will be identified and categorised using validated vertical axis cut-points in unit counts per minute (cpm): sedentary behaviour (0–184 cpm), light (185–2424 cpm), moderate (2425–3268 cpm) and vigorous physical activity (≥3269 cpm).^40^

All data processing procedures will follow the recommendations by Migueles *et al* and the protocols of the motoric module of the nationwide, representative surveys on child and adolescent health.^39 41^ The datasets were considered valid based on international standards, requiring ≥8 hours of wear time on ≥4 weekdays and ≥1 weekend day.^42^ If there are fewer than 5 days with usable data, the participants will be asked to repeat the accelerometry. If still less than five valid days are provided, participants will be excluded from the analyses due to non-adherence.

### Patient-reported outcome measures

Health-related quality of life refers to the current health situation in relation to personal expectations and will be assessed on the basis of patient-reported outcomes. The measurement of health-related quality of life will allow to assess the patient’s state of health, social functioning, mental health and well-being and will be measured at baseline and 12 months, to assess changes in health-related quality of life.

Health-related quality of life will be assessed using the EuroQol 5-Dimension 5-Level questionnaire in its version for adults (EQ-5D-5L) and the youth-specific version for children and adolescents (EQ-5D-Y-5L), which is a validated instrument that enables a comparison of health status across different patients, disease areas and treatments.^43 44^ The principal secondary outcome of the health-related quality of life questionnaires will be the EuroQol 5-Dimension (EQ5D) index which will be calculated by converting a person’s responses to the five-dimensional EQ5D questions into a single summary score using a country-specific value set.^45^ Patients with haemophilia will also complete the questionnaire on subjective physical function in patients with haemophilia, the Haemophilia & Exercise Project-Test-Questionnaire (Hep-Test-Q), and the questionnaire on quality of life in haemophilia, the Haemophilia quality-of-life questionnaire (Haemo-QoL).^46^,^49^ Additionally, activity habits from all participants will be collected questionnaire-based using the activity questionnaire Motorik-Module Activity Questionnaire (MoMo-AFB).^50^

### Interim analysis

Six months after the initial assessment, an interim analysis will be conducted. The results from the video gait analysis and accelerometry in patients with haemophilia will be reviewed by an interdisciplinary team, and individualised recommendations for optimising joint health will be provided to each patient alongside their assessment results. Healthy controls will be notified of video gait analysis results only if abnormal findings are detected. However, they will receive feedback on their accelerometry-measured daily activity in comparison with age-specific WHO recommendations.^51^ The authors acknowledge that conducting an interim analysis at 6 months may influence outcomes at 12 months and may limit the interpretability of longitudinal findings. Nonetheless, in the role of clinicians, and in light of the potential long-term consequences of unaddressed early gait pathology, the authors consider it an ethical obligation to respond promptly to any clinically relevant abnormalities which will be identified. Such actions will not be considered part of the study intervention but will be regarded as standard ethical clinical care.

### Statistical methods

Statistical analyses will be performed using SPSS Statistics 31.0.0 (IBM Corporation, Armonk, NY, USA). Feasibility as the primary outcome of this pilot study will be evaluated by calculating the percentage of people contacted (recruitment rate) and those who completed all assessments (refusal, retention and drop-out rate). The proportion of usable video gait recordings, participant adherence to accelerometry, proportion of missing data and questionnaire completion rate will be summarised. Reasons for ineligibility or refusal will be documented and interpreted by the research team to identify relevant topics (table 2). Data will be summarised using frequencies, percentages, means, SD, medians and ranges, as appropriate. CIs (95%) will be calculated for key feasibility parameters (eg, recruitment and retention rates) to estimate the precision of these outcomes. Patterns of missing data will be examined and reported.

As this is a feasibility study, participant characteristics and outcome measures will be examined for normality and summarised using appropriate tests. A table will be included to compare key demographic and clinical characteristics between the two patient groups and the healthy participants. Variance, SD and differences between the patient groups and the healthy participants in spatiotemporal and kinematic gait parameters, as well as in the HJHS, will be reported. Changes over time (baseline, follow-up and change from baseline) will also be calculated and compared. No a priori hypotheses will be tested; therefore, all p values are reported as descriptive measures. Associations and potential influences of age, physical activity and health-related quality of life on gait parameters and HJHS will be reported. These variables will either be included as covariates in the statistical analyses or explored through subgroup analysis, as appropriate. Where applicable, comparisons with age-specific healthy participants and reference values from the literature will be made.

Given the use of multiple questionnaires and outcome measures, we acknowledge the increased risk of type I error due to multiple comparisons. However, the use of various questionnaires will allow for a more nuanced interpretation of the results and help identify the most meaningful and practical instruments for this cohort in future studies. As this is a pilot feasibility study, all findings will be considered exploratory rather than confirmatory and interpreted with caution.

Based on the literature, the most suitable candidate for evaluating the potential benefits of our programme will be the difference in spatiotemporal gait parameters between the two groups. Previous studies have reported decreased gait speed, shorter step length and reduced range of motion in the knee and hip joints among individuals with haemophilia, even in the absence of clinically evident arthropathy.^7 52^ Reported median gait speeds for healthy individuals from toddlers to adults range from 0.9 to 1.4 m/s, depending on age.^53^,^56^ Normalisation to body height,^57^ and comparison with normative data collected within the same laboratory will help reduce age-related variability. Currently, no comparable data exists in individuals with haemophilia across the entire age range from 18 months to 30 years. Therefore, this pilot study is designed to obtain valid and robust estimates of spatiotemporal and kinematic gait parameters in this population.

### Limitations

The present study protocol has some limitations. First, this study is limited by its feasibility single-centre design with a rather small cohort size, limiting the ability to generalise the results. Moreover, the study consists of several different parts with a complex design, meaning additional workload for the patients. Hence, the willingness of patients to participate in the study is unclear. When using questionnaires to assess activity and quality of life, a response bias cannot be ruled out completely. Besides, the quality of the accelerometry results is dependent on the adherence of patients to the study protocol, especially since data collection will take place unsupervised. Finally, the main limitation is its limited statistical power and interpretability due to heterogeneity of the cohort including participants of a wide age range.

Notwithstanding these limitations, the primary objective of this pilot feasibility study is to optimise the study protocol, improve recruitment and retention strategies, and refine data collection methodologies in advance of conducting adequately powered, definitive trials. Thus, feasibility outcomes are the primary endpoints rather than statistically significant clinical differences.

However, there are several mitigating strategies included in the protocol to reduce the limitation factors. To reduce the workload for the patient the assessment is included in the haemophilia check-up appointment. Thus, no extra travel time is needed. To support adherence, the participants will receive a small participation compensation after completing the assessment and handing in the accelerometer. The clinical counselling after 6 months can facilitate recruitment and adherence. For bias reduction, only validated questionnaires will be used.

### Ethics and dissemination

Written informed consent will be obtained from all caregivers of the participants and informed assent or consent from the patients and participants depending on the age at study inclusion. All measurements obtained during the study will be safe and not harmful. The examination can be terminated at any time at the patient’s request.

The study results may inform individual clinical counselling for participants with haemophilia. However, no direct benefit is expected for individuals with haemophilia or those in the control group.

With regard to the risk assessment of the project, there is a calculable data protection risk due to the video recording and the use of the videos to generate an evaluation algorithm. All data will be collected, processed and pseudonymised and stored in accordance with the rules of Good Clinical Practice and will only be accessible to authorised employees who will be directly involved in the research project. The storage and evaluation of this study-related data will be carried out in accordance with legal regulations. All steps will be carried out at the University of Tübingen and no individual data will be shared with third parties.

The research will be conducted in accordance with the Declaration of Helsinki and has been approved by the Ethics Committee at the Medical Faculty and the University Hospital Tübingen (project No. 188/2023BO1, approval 5 July 2023, protocol version 2.1).

Study results will be disseminated through national and international scientific conferences and publication in open-access, peer-reviewed journals. Authorship will follow the DFG ‘Guidelines for Safeguarding Good Research Practice’.

## Discussion

This pilot study aims to assess the feasibility, practicability and acceptability of the proposed study design, data collection procedures and outcome measures in preparation for a future definitive trial.

To the best of our knowledge, this is the first published study protocol describing a pilot feasibility study incorporating a longitudinal design and including age-matched healthy controls to conduct multicomponent qualitative and quantitative motion analysis in young patients with haemophilia.

Consistent with the aims of pilot and feasibility studies, the study is not designed to evaluate clinical effectiveness, and any longitudinal changes observed will be analysed and reported descriptively and will not be interpreted as evidence of intervention effects.

For the first time, a markerless video-based gait analysis will be applied to patients with haemophilia. While 3D gait analysis has previously been shown to detect early functional changes in this patient group,^17 19 58^ it remains resource-intensive and impractical for routine clinical use. By evaluating markerless, AI-driven video gait analysis combined with accelerometry and clinical scores, this study bridges the gap between highly specialised motion analysis methods and routine clinical monitoring in haemophilia patients.

The study will also integrate both self-reported and objectively measured physical activity using accelerometry. Activity decreases with increasing joint problems; patients with mild arthropathy are still active but with lower intensity.^59^ Given the known limitations of questionnaire-based assessments, particularly in children,^41^ the parallel collection of objective data may improve accuracy and provide insight into everyday activity levels. Additionally, health-related quality of life is included in the study protocol, acknowledging its relevance in chronic disease monitoring. According to a recent review, previous studies show a very heterogeneous quality of life in patients with haemophilia between 2010 and 2021, however, emerging evidence suggests that effective prophylactic treatment improves quality of life.^60^

The integration of joint function, physical activity and quality of life into a composite outcome framework will reflect the multidimensional nature of haemophilia-related morbidity.
