# Supplementary material for: Quantitative and qualitative motion analysis for early detection of joint damage in haemophilia patients: study protocol for a pilot feasibility study at a German haemophilia treatment centre
Source: BMJ Open. 2026 Mar 31;16(3):e108291. doi: 10.1136/bmjopen-2025-108291 (PMC13052583; doi:10.1136/bmjopen-2025-108291)
Supplement: Supplementary file 1 [file bmjopen-2025-108291.reviewer_comments.pdf]

PEER REVIEW HISTORY

BMJ Open publishes all reviews undertaken for accepted manuscripts. Reviewers are asked to complete a checklist review form and are provided with free text boxes to elaborate on their assessment. These free text comments are reproduced below.

ARTICLE DETAILS

Title (Provisional)

Quantitative and qualitative motion analysis for early detection of joint damage in haemophilia patients – Study protocol for a pilot feasibility study

Authors

Schober, Sarah; Hansmann, Sandra

VERSION 1 - REVIEW

**Reviewer**	**1**
**Name**	**Cuesta-Barriuso, Rubén**
**Affiliation**	**University of Oviedo, Department of Surgery and Medical-Surgical Specialties**
**Date**	**25-Aug-2025**
**COI**	**None**

I would like to thank the authors for the opportunity to review this manuscript. The study protocol presented addresses a highly relevant and timely issue in haemophilia care: the early detection of joint impairment through the integration of artificial intelligence-based marker-less gait analysis with established clinical and patient-reported outcomes. The integration of artificial intelligence-driven marker-less gait analysis with established clinical tools (HJHS, accelerometry, QoL measures) is innovative and has clear potential to advance early detection of haemophilic arthropathy. This is an innovative and ambitious project, and I commend the authors for their efforts to combine quantitative and qualitative methods within a longitudinal feasibility design. The focus on improving early detection and potentially informing personalised management strategies represents a valuable contribution to the field.

That being said, there are several important aspects of the manuscript that require clarification and refinement before the work can be considered for publication in a leading journal. My review aims to provide constructive feedback, focusing particularly on methodological rigour, clarity of reporting, and the need to reduce speculative or promotional language. I have structured my comments by section so that the authors can readily identify where improvements are required.

Abstract

- The abstract is overly long (402 words). BMJ Open guidelines recommend concision. Consider streamlining by removing background detail already expanded in the Introduction.

- Methods: the description of "marker-less video motion analysis" is vague. Readers should know which neural network architecture or training approach will be used, and how outcomes will be quantified.

- The statement "The data will directly benefit the participants…" is more suited to the ethics section than the abstract.

- Phrasing such as "to make a sustainable contribution" is too promotional. Abstracts should remain strictly factual.

Introduction

- Overall the introduction is comprehensive, but somewhat repetitive. Multiple sections reiterate the role of physical activity and the importance of early joint monitoring. This could be shortened without loss of clarity.

- Page 6, line 95: "Introcuction" should be corrected to "Introduction."

- The review of prior literature on 3D gait analysis is adequate, but the gap statement is not fully compelling. The authors must justify why marker-less analysis is superior beyond cost and accessibility—what is known about validity, reliability, and clinical interpretability?

- The aim and objectives are clearly stated, but would benefit from sharper articulation. Currently, they read more like aspirations than precise research questions. For example: "The primary aim is to explore usability, acceptability, and reliability…" could be restructured into a specific testable hypothesis.

Methods

This is the section most in need of refinement.

Study design

- The design is described as "observational hypothesis-generating pilot study." However, "pilot feasibility study" would be more precise. Explicit criteria for feasibility (e.g., recruitment rates, adherence, data completeness) should be defined.

- The inclusion of both children and young adults (>18 months to <30 years) creates a very broad heterogenous cohort. How will age-related variability in gait and physical activity be managed analytically?

Sample size

- The justification ("12 subjects per group is sufficient for pilot studies") is weak. While this is a common rule of thumb, feasibility studies should clearly state the parameters being

estimated to inform future sample size calculations. At present, the rationale appears arbitrary.

- Power calculations are absent. Even for feasibility work, the authors should state what minimal detectable effect size they are expecting to observe.

Recruitment

- Restriction to a single centre is acknowledged, but recruitment feasibility is not discussed. What is the expected pool of eligible patients at Tuebingen? Is the recruitment window realistic?

Interventions / assessments

- The description of gait analysis is detailed but still leaves uncertainties. For example:

- How many walking trials per participant?

- How will variability between trials be managed?

- Which outcome parameters are pre-specified as primary (e.g., cadence, joint angles)?

- Accelerometry: the authors state they will require ≥5 days of valid wear. This is standard, but how will non-adherence be handled? Will missing days be imputed?

- Questionnaires: several instruments are listed (MoMo-AFB, EQ-5D, Haemo-QoL, HEP-Test Q). It is unclear which is considered the principal secondary outcome. Multiple comparisons risk should be addressed.

- Interim analysis at 6 months (providing feedback to patients) may influence outcomes at 12 months. This introduces a quasi-interventional element that complicates interpretation. Authors should clarify whether this is intended as part of the intervention or merely ethical patient care.

Statistical analysis

- The plan lacks clarity. Listing numerous tests (t-test, Wilcoxon, Chi-square, regression, Bland-Altman, etc.) without specifying which variables they will be applied to risks appearing unfocused.

- The choice between parametric and non-parametric approaches should be based on clear pre-specified criteria.

- No adjustment for multiple comparisons is described.

- As this is a pilot study, emphasis should be on descriptive statistics and estimation (confidence intervals) rather than hypothesis testing.

Discussion

- The discussion is too speculative for a protocol paper. Phrases such as “This could form the basis for future diagnostic testing” and “may enable personalised medicine” read as promotional rather than strictly methodological reflection.

- Limitations are acknowledged (small cohort, monocentric design, workload for patients, potential bias in self-reports, accelerometer adherence). However, these should be presented earlier, with explicit mitigation strategies.

- The discussion of potential real-world impact should be reduced, and instead focus on how the protocol addresses gaps in methodology.

Study limitations

- These are appropriate but underdeveloped. The most serious limitation—the possible lack of statistical power and interpretability due to heterogeneity—is not adequately highlighted.

- The authors should clarify whether the feasibility outcomes (recruitment, adherence, data quality) are the primary endpoints, rather than clinical differences per se.

Conclusions

- Conclusions should be removed or drastically shortened in a protocol. A protocol should not imply that the study will succeed but rather that it will address specific feasibility questions.

References

- Generally comprehensive, but some citations are dated. For example, reliance on Julious (2005) for sample size justification is outdated. More recent guidance on pilot trial design should be cited.

- Reference formatting needs minor consistency checks (spacing, punctuation).

Tables and Figures

- Table 1 (study characteristics) is clear, but overly dense. Consider splitting into separate tables for eligibility criteria and interventions.

- Table 2 (tools and categories) is helpful but could be structured more clearly with subheadings.

- Figures 1–3 are schematic but add little beyond text description. They should be simplified for clarity.

- In Figure 2, “step lenght” should be corrected to “step length.”

Specific Language/Stylistic Issues

- The manuscript requires careful proofreading. Examples include:

- p.6: “Introcuction” → “Introduction.”

- p.20: “Therefor” → “Therefore.”

- “proband” is used repeatedly; “participant” is more standard in medical English.

- Tense is inconsistent; protocols should primarily use future tense (“will be assessed”).

- Some sentences are overly promotional (e.g., “make a sustainable contribution”). Replace with neutral, scientific phrasing

---

VERSION 1 - AUTHOR RESPONSE

Reviewer #1

Abstract

1. **Length: The abstract is overly long (402 words). BMJ Open guidelines recommend concision. Consider streamlining by removing background detail already expanded in the Introduction.**

   Thank you for this comment. We revised and shortened the abstract considerably and removed the majority of the background details. The total word count of the abstract was reduced from 402 to 291 words (p. 2, l. 21 - p.3, l.46).

2. **Methods: the description of “marker-less video motion analysis” is vague. Readers should know which neural network architecture or training approach will be used, and how outcomes will be quantified.**

   We thank the reviewer for bringing this point to our attention. A detailed description of the used neural network architecture and training approach of the marker-less video motion analysis was added in the *gait analysis* paragraph (p. 12, l. 204- l. 218). Furthermore, the quantification of outcomes is explained at the end of this segment (p. 12, l. 219-p.13, l. 224). The neural network architecture and training approach, as well as the methods for quantifying outcomes, are highly complex and therefore require a detailed explanation. For this reason, the authors chose not to include this information in the abstract but to describe it comprehensively in the *methods and analysis* section.

3. **Statement and phrasing: The statement “The data will directly benefit the participants…” is more suited to the ethics section than the abstract. Phrasing such as “to make a sustainable contribution” is too promotional. Abstracts should remain strictly factual.**

Indeed there was some unfortunate phrasing in the abstract. We have thoroughly revised the abstract. The statements “The data will directly benefit the participants…” and the “to make a sustainable contribution” was removed from the abstract and replaced by more appropriate, fact-based language (p. 2, l. 21 - p.3, l.46).

Introduction

1. **Length and clarity: Overall the introduction is comprehensive, but somewhat repetitive. Multiple sections reiterate the role of physical activity and the importance of early joint monitoring. This could be shortened without loss of clarity.**

   Thank you for this comment. We revised the introduction and removed all redundant and repetitive elements resulting in a shorter and more concise introduction (p.4, l. 61 - p.5, l.105).

2. **Misspelling: Page 6, line 95: “Introcuction” should be corrected to “Introduction.”**

   We apologies for the misspelling of introduction. The spelling has been corrected in the revised version of the manuscript (p.4, l. 61).

3. **Gap statement: The review of prior literature on 3D gait analysis is adequate, but the gap statement is not fully compelling. The authors must justify why marker-less analysis is superior beyond cost and accessibility—what is known about validity, reliability, and clinical interpretability?**

   We are grateful for the reviewer’s feedback and included a new section explaining the superiority and importance of the marker-less gait analysis in the revised manuscript. The new section focusses on validity, reliability, and clinical interpretability of the new method (p.4 ,l. 79 – p. 5, l. 87).

4. **The aim and objectives are clearly stated, but would benefit from sharper articulation.**

   We appreciate the reviewer’s comment and revised the aim and objective section to clarify the precise research questions. The aim of the pilot feasibility

study is to test feasibility, practicability and acceptance of the procedures for a quantitative and qualitative motion analysis for early detection of joint damage in haemophilia. The primary objectives, exploratory primary objectives and secondary objectives are described in the *aim and objective* paragraph (p.5, l. 107 - p. 6, l. 121).

Methods

Study design

1. **Pilot feasibility study: The design is described as "observational hypothesis-generating pilot study." However, "pilot feasibility study" would be more precise. Explicit criteria for feasibility (e.g., recruitment rates, adherence, data completeness) should be defined.**

   Following the reviewer's recommendation we have changed the description of the study design from "observational hypothesis-generating pilot study" to "pilot feasibility study". Besides, we defined explicit criteria for feasibility such as recruitment rates, adherence, data completeness etc. (p.6, l. 124-129, and table 2).

2. **Age-related variability in gait and physical activity: The inclusion of both children and young adults (>18 months to <30 years) creates a very broad heterogenous cohort. How will age-related variability in gait and physical activity be managed analytically?**

   We have considered the reviewer's point carefully. Analytically we will manage the age-related variability by three different approaches. First of all, we will include age-matched healthy controls and additionally comparisons will be made against age-specific reference values from the literature, as appropriate. Furthermore, gait data will be normalized to participants height, age-specific cut-points for accelerometer data, and age-appropriate questionnaires will be used. Finally, participants might be divided into age groups for subgroup analysis if applicable. This new aspects have been added to the revised manuscript in the *participants and recruitment* and *statistical methods* section (p. 8, l. 154-155 and p. 16, l. 299 – 304 and l.314 -316).

Sample Size

1. **The justification ("12 subjects per group is sufficient for pilot studies") is weak. While this is a common rule of thumb, feasibility studies should clearly state the parameters being estimated to inform future sample size calculations.**

   The reviewer raises a valid point. Methodological recommendations for feasibility studies suggest that samples of 12–30 participants per group are sufficient to assess feasibility outcomes such as recruitment rates, retention, data completeness and preliminary parameter estimates for future sample size calculations (*Julious et al., 2005; Lancaster et al.2004, Eldridge et al. 2016*) (p. 8, l.139-146).

   The sample size for this feasibility pilot study is primarily guided by practical considerations, including participant flow, budgetary constraints, and the number of participants needed to reasonably evaluate feasibility objectives (*Teresei et al*., *2022*). Given that hemophilia is a rare disease, recruiting a large sample of patients with severe hemophilia and the same number of patients with moderate haemophilia at a single centre is generally challenging. The study is designed to assess feasibility aspects such as recruitment rates, retention, and adherence, rather than to detect statistically significant differences between groups. Therefore, we are confident that the feasibility assessment can be achieved with a relatively small sample size of 12 participants for each group.

   After consultation with our statistician, a sample size calculation was performed based on the difference in gait speed between rheumatic patients and healthy controls reported by Vincent et al. (2022). A sample size of 21 participants per group (severe haemophilia, moderate haemophilia, and healthy controls) would provide 80% power to detect a mean difference of 0.2 m/s, assuming a common standard deviation of 0.2 m/s and a significance level of 5%. However, we expect the difference between groups to be smaller, as two types of haemophilia will be compared. In addition, a larger standard deviation is anticipated due to the wide age range of participants. Therefore, we consider that a larger sample size will be required for a future trial, and a multicenter approach will be necessary.

2. **Power calculation: Power calculations are absent. Even for feasibility work, the authors should state what minimal detectable effect size they are expecting to observe.**

   Thank you for pointing out the issue regarding power calculation. The primary aim of this pilot feasibility study is not to test hypotheses or detect statistically significant differences between groups. Therefore, a conventional power calculation is generally not practicable. Instead, the focus is on assessing feasibility aspects such as recruitment rates, retention and adherence. A small, pragmatic sample is chosen to allow estimation of variability and standard deviations, which can then inform the sample size and power calculation for a subsequent larger definitive trial. For future sample size calculation for a multicenter study a power of 0.8 was estimated (see section *sample size* above).

Recruitment

1. **Recruitment feasibility: Recruitment feasibility is not discussed. What is the expected pool of eligible patients at Tuebingen? Is the recruitment window realistic?**

   In response to the reviewer's suggestion a new paragraph (in *participants and recruitment*) explaining the expected pool of eligible patients in Tuebingen and recruitment feasibility was incorporated in the manuscript and the estimated recruitment window was added to the paragraph *timeline* (p.8, l. 139 -148 and p. 9, l. 160-162)

Interventions/assessments

1. **Gait analysis: The description of gait analysis is detailed but still leaves uncertainties. For example:**

   - How many walking trials per participant?

   - How will variability between trials be managed?

   - Which outcome parameters are pre-specified as primary (e.g., cadence, joint angles)?

   Following the reviewer's recommendation, we have added information such as walking trials per participant, management of variability between trials and pre-specified primary outcome parameters. Additionally, details of the developed

qualitative, markerless gait analysis and specifications of the gait laboratory are inserted (p.11, l. 191 – p.13, l. 225).

2. **Accelerometry: the authors state they will require ≥5 days of valid wear. This is standard, but how will non-adherence be handled? Will missing days be imputed?**

   As suggested, we have clarified how we will manage non-adherence and missing days. Our study follows the protocol of the international children's accelerometry database (ICAD) *(Sherar et al., 2011)*: If there will be less than five days with usable data, the participants will be asked to repeat the accelerometry. If they still won't manage to provide five valid days, participants will be excluded from the analyses due to non-adherence (p. 14 l. 249 – 254).

3. **Questionnaires: several instruments are listed (MoMo-AFB, EQ-5D, Haemo-QoL, HEP-Test Q). It is unclear which is considered the principal secondary outcome. Multiple comparisons risk should be addressed.**

   We thank the reviewer for this helpful suggestion and added the principal secondary outcome and added a section addressing multiple comparisons risk in the *statistical analysis* paragraph.

   The principal secondary outcome of the health-related quality of life questionnaires will be the EQ5D index which will be calculated by converting a person's responses to the 5 dimensions EQ5D questions into a single summary score using a country-specific value set (*Kreimeier et al., 2022*). The principle secondary outcome will be measured at baseline and 12 months, to assess health-related quality of life (p.14, l. 261-266).

   Given the use of multiple questionnaires and outcome measures, we recognize the increased risk of Type I error due to multiple comparisons. However, the use of various questionnaires will allow for a more nuanced interpretation of the results, and help identify the most meaningful and practical instruments for this cohort in future studies. As this is a pilot feasibility study all findings will be considered exploratory rather than confirmatory, and interpreted with caution (p.16, l. 305–309).

4. **Interim analysis: at 6 months (providing feedback to patients) may influence outcomes at 12 months. This introduces a quasi-interventional element that complicates interpretation. Authors should clarify whether this is intended as part of the intervention or merely ethical patient care.**

   We appreciate the reviewer's insightful comment. A new section explaining the interim analysis was integrated in the revised manuscript. The authors acknowledge that conducting an interim analysis at six months may influence outcomes assessed at 12 months and may limit the interpretability of longitudinal findings. Nonetheless, in our role as clinicians, and in light of the potential long-term consequences of unaddressed early gait pathology, we consider it an ethical obligation to respond promptly to any clinically relevant abnormalities which will be identified. Such actions will not be considered part of the study intervention but will be regarded as standard ethical clinical care (p. 14, l. 270 – 282).

Statistical analysis

1. **Tests: The plan lacks clarity. Listing numerous tests (t-test, Wilcoxon, Chi-square, regression, Bland-Altman, etc.) without specifying which variables they will be applied to risks appearing unfocused.**

   In response to the reviewer's suggestion we have clarified the statistical methods which will be used. Statistical analyses will be primarily descriptive, focusing on feasibility outcomes such as recruitment rates, reasons for ineligibility or refusal, retention rate, drop-out rate, proportion of usable video gait recordings, participant adherence to accelerometry, proportion of missing data, questionnaire completion rate. Data will be summarized using frequencies, percentages, means, standard deviations, medians, and ranges, as appropriate. Confidence intervals (95%) will be calculated for key feasibility parameters (e.g., recruitment and retention rates) to inform the precision of these estimates. Missing data patterns will be explored and reported (p.15, l. 283 – p.16, l. 303).

2. **Parametric and non-parametric approaches: The choice between parametric and non-parametric approaches should be based on clear pre-specified criteria.**

The comment is well taken. Prior to selecting parametric or non-parametric approaches the data will be examined for normality and analyzed accordingly (p. 15, l. 294 – 295).

3. **No Adjustment for multiple comparison is described**

   In line with the reviewer's comment, we recognize the increased risk of type I error due to multiple comparisons. We address this topic in the *section interventions and assessments 3* of this letter. A more detailed description is provided in that section.

4. **As this is a pilot study, emphasis should be on descriptive statistics and estimation (confidence intervals) rather than hypothesis testing.**

   We thanks the reviewer for the constructive remark. Details on the planned descriptive statistics and estimations are provided in *paragraph 1 of the statistical analysis section* of this letter.

Discussion

1. **Too speculative: The discussion is too speculative for a protocol paper. Phrases such as "This could form the basis for future diagnostic testing" and "may enable personalized medicine" read as promotional rather than strictly methodological reflection.**

   We recognize the importance of this point and have addressed it thoroughly by revising the discussion and eliminating all speculative aspects and focusing on a strictly methodological reflection (p.18, l. 363 – p.19, l. 393).

2. **Limitations: Limitations are acknowledged (small cohort, monocentric design, workload for patients, potential bias in self-reports, accelerometer adherence). However, these should be presented earlier, with explicit mitigation strategies. The most serious limitation—the possible lack of statistical power and interpretability due to heterogeneity—is not adequately highlighted.**

   Following the reviewer's recommendation, we have elaborated on this point in the revised version adding mitigation strategies to the limitations and positioning the section earlier in the manuscript: right after *statistical methods*

and before *ethics and dissemination*. Furthermore we emphasized the possible lack of statistical power and interpretability due to heterogeneity (p. 16, l. 320 – p.17, l. 340).

3. **Focus on addressing gaps in methodology: The discussion of potential real-world impact should be reduced, and instead focus on how the protocol addresses gaps in methodology.**
   The reviewer is correct, and we have decreased the discussion of potential real-world impact focusing more on the methodologic relevance of the study.

4. **Primary endpoints: The authors should clarify whether the feasibility outcomes (recruitment, adherence, data quality) are the primary endpoints, rather than clinical differences per se.**
   We agree that clarification was needed in this section, and we have now revised the text of the discussion accordingly. Feasibility outcomes are clearly stated as the primary endpoints.
   The primary objectives, exploratory primary objectives and secondary objectives are described in the *aim and objective* paragraph (p.5, l. 107-p. 6, l. 121, table 1).

Conclusion

1. **Shortened or removed: Conclusions should be removed or drastically shortened in a protocol. A protocol should not imply that the study will succeed but rather that it will address specific feasibility questions.**
   We concur with the reviewer's assessment and have removed the conclusion statement from the manuscript.

References

1. **More modern data: Generally comprehensive, but some citations are dated. More recent guidance on pilot trial design should be cited.**
   Following the reviewer's recommendation we have updated the reference list and have focused on more recent data (see references section). With regards to pilot trial design more recent data was added (*Eldridge et al.,2016*; *Teresi et al., 2022*).

2. **Formatting: Reference formatting needs minor consistency checks (spacing, punctuation)**
   Thank you for the comment. We revised the reference formatting carefully (p.20 l. 394 – p. 23, l. 549).

Tables and Figures

1. **Table 1: (study characteristics) is clear, but overly dense. Consider splitting into separate tables for eligibility criteria and interventions.**
   We are grateful for this helpful suggestion. We have revised table 1 and included subheadings for a clearer overview (*study design, eligibility and interventions*) (see table 1).

2. **Table 2: tools and categories is helpful but could be structured more clearly with subheadings.**
   As suggested, we have added four new subheadings (*Feasibility measurement data, Clinical measurement data*, *Medical record data* and *Patient reported outcome measures*) in table 2 (see table 2).

3. **Figures 1-3: Figures 1–3 are schematic but add little beyond text description. They should be simplified for clarity.**
   In line with the reviewer's comment, we have now modified and clarified the figures 1-3.

4. **Figure 2: "step length" should be corrected to "step length"**
   We apologize for the misspelling of step length which has been corrected in the revised manuscript.

Specific Language/Stylistic Issues

1. **Proofreading: - The manuscript requires careful proofreading**
   Thank you for this comment. We have carefully proofread the revised manuscript.

2. **Misspelling of introduction and therefore: p.6: “Introcuction” → “Introduction.”, p.20: “Therefor” → “Therefore.”**
   We are sorry for these mistakes and corrected the misspelling in the revised manuscript.

3. **Participant: “proband” is used repeatedly; “participant” is more standard in medical English.**
   We thank the reviewer for drawing our attention to this and have changed probands to participant in all the manuscript.

4. **Future tense: Tense is inconsistent; protocols should primarily use future tense (“will be assessed”).**
   We thank the reviewer for highlighting this and have corrected all tense inconsistencies to future tense in the manuscript.

5. **Promotional language: Some sentences are overly promotional (e.g., “make a sustainable contribution”). Replace with neutral, scientific phrasing**
   We recognize the importance of this point and have addressed it thoroughly in the revised manuscript by using a neutral and objective language.

We would like to thank you and the editor and reviewer once again for the thorough review and the constructive and helpful comments. We think that the manuscript was enhanced considerably. We hope that we have thoroughly addressed all the issues raised by the reviewer, we would be delighted if the manuscript is deemed suitable for publication in *BMJOpen.*

VERSION 2 - REVIEW

**Reviewer**	**1**
**Name**	**Cuesta-Barriuso, Rubén**
**Affiliation**	**University of Oviedo, Department of Surgery and Medical-Surgical Specialties**
**Date**	**12-Dec-2025**

COI

I would like to commend the authors for the revisions undertaken. The manuscript has improved substantially compared with the previous version and addresses the reviewers' comments in a clear and verifiable manner. The study is now appropriately framed as a pilot feasibility study, with a coherent distinction between feasibility-related primary objectives and exploratory clinical outcomes. The methodological description of the markerless gait analysis has been considerably strengthened, the sample size is now adequately justified for a feasibility design, and the statistical analysis plan is appropriately aligned with the exploratory nature of the study. Overall, the protocol currently demonstrates good methodological rigour, internal coherence, and clarity, and is well suited to the scope of BMJ Open.

Nevertheless, prior to final acceptance, a small number of minor issues should be addressed. These relate primarily to conceptual clarity and interpretative emphasis and do not require any changes to the study design.

Minor comments

Terminology of study objectives

- The term "exploratory primary objectives" is not commonly used in methodological reporting and may cause confusion. The authors are encouraged to replace this wording with a more standard term (e.g. "exploratory outcomes" or "exploratory clinical outcomes"), while maintaining a clear statement that feasibility outcomes constitute the sole primary objectives of the study.

Interpretation of the interim analysis

- Although the interim analysis at six months is appropriately justified on ethical grounds, it would be helpful to explicitly reiterate in the Discussion that any longitudinal changes observed should be interpreted descriptively and not as intervention effects.

Ethical wording regarding direct benefit

- In the Ethics section, there remains a reference to potential direct benefit for participants. For a study protocol, this wording would benefit from further neutralisation (e.g. indicating that results may inform individual clinical counselling rather than implying direct benefit).

Minor condensation of the final discussion

- The final part of the Discussion could be slightly condensed to reduce redundancy and to maintain a clear focus on the methodological contribution of the protocol rather than its potential future impact.

VERSION 2 - AUTHOR RESPONSE

Reviewer #1

1. **Terminology of study objectives**

   The term "exploratory primary objectives" is not commonly used in methodological reporting and may cause confusion. The authors are encouraged to replace this wording with a more standard term (e.g. "exploratory outcomes" or "exploratory clinical outcomes"), while maintaining a clear statement that feasibility outcomes constitute the sole primary objectives of the study.

   We thank the reviewer for this constructive comment and fully agree. The suggested change has been incorporated into the revised manuscript – "exploratory primary objectives has been replaced by "exploratory clinical outcomes" or "exploratory outcomes", as appropriate. Additionally, a new sentences was added, stating that "this clinical trial is designed with feasibility outcomes as the only primary objectives. All clinical, outcomes are considered exploratory" (p. 6, l. 117 – 121, p. 13. L. 223, and Table 1).

2. **Interpretation of the interim analysis**

   Although the interim analysis at six months is appropriately justified on ethical grounds, it would be helpful to explicitly reiterate in the Discussion that any longitudinal changes observed should be interpreted descriptively and not as intervention effects.

   We appreciate the reviewer's suggestion and have implemented the change in the discussion section (p. 18, l. 368 – p. 19, l. 370).

3. **Ethical wording regarding direct benefit**

   In the Ethics section, there remains a reference to potential direct benefit for participants. For a study protocol, this wording would benefit from further neutralisation (e.g. indicating that results may inform individual clinical counselling rather than implying direct benefit).

   We acknowledge the reviewer's point and have incorporated a clarification in the manuscript explaining that there will be no direct benefit for any participant of the study. Nevertheless, the study results may inform individual clinical counselling (p. 17, l. 338 and p. 18, l. 345 – 346).

4. **Minor condensation of the final discussion**

The final part of the Discussion could be slightly condensed to reduce redundancy and to maintain a clear focus on the methodological contribution of the protocol rather than its potential future impact.

We concur with the reviewer's assessment and have revised the discussion section and removed the final part, resulting in a more concise and methodological-focused discussion (p. 18, l. 365 – p. 19, l. 386).

We thank you, the editor, and the reviewer once again for the careful evaluation of our manuscript and for the constructive and helpful comments. We trust that all issues raised have been adequately addressed and would appreciate the manuscript being considered suitable for publication in *BMJ Open*.
